# Influence of the Anthocyanin and Cofactor Structure on the Formation Efficiency of Naturally Derived Pyranoanthocyanins

**DOI:** 10.3390/ijms22136708

**Published:** 2021-06-23

**Authors:** Gonzalo Miyagusuku-Cruzado, Danielle M. Voss, M. Monica Giusti

**Affiliations:** Department of Food Science and Technology, The Ohio State University, 2015 Fyffe Rd., Columbus, OH 43210 1007, USA; miyagusukucruzado.1@osu.edu (G.M.-C.); voss.129@osu.edu (D.M.V.)

**Keywords:** hydroxyphenyl-pyranoanthocyanins, naturally derived pigments, accelerated formation, 10-catechyl-pyranoanthocyanins

## Abstract

Pyranoanthocyanins are anthocyanin-derived pigments with higher stability to pH and storage. However, their slow formation and scarcity in nature hinder their industrial application. Pyranoanthocyanin formation can be accelerated by selecting anthocyanin substitutions, cofactor concentrations, and temperature. Limited information is available on the impacts of the chemical structure of the cofactor and anthocyanin; therefore, we evaluated their impacts on pyranoanthocyanin formation efficiency under conditions reported as favorable for the reaction. Different cofactors were evaluated including pyruvic acid, acetone, and hydroxycinnamic acids (*p*-coumaric, caffeic, ferulic, and sinapic acid) by incubating them with anthocyanins in a molar ratio of 1:30 (anthocyanin:cofactor), pH 3.1, and 45 °C. The impact of the anthocyanin aglycone was evaluated by incubating delphinidin, cyanidin, petunidin, or malvidin derivatives with the most efficient cofactor (caffeic acid) under identical conditions. Pigments were identified using UHPLC-PDA and tandem mass spectrometry, and pyranoanthocyanin formation was monitored for up to 72 h. Pyranoanthocyanin yields were the highest with caffeic acid (~17% at 72 h, *p* < 0.05). When comparing anthocyanins, malvidin-3-*O*-glycosides yielded twice as many pyranoanthocyanins after 24 h (~20%, *p* < 0.01) as cyanidin-3-*O*-glycosides. Petunidin- and delphinidin-3-*O*-glycosides yielded <2% pyranoanthocyanins. This study demonstrated the importance of anthocyanin and cofactor selection in pyranoanthocyanin production.

## 1. Introduction

Anthocyanins (ACNs) are dietary flavonoids with bright colors that can range from red to blue [1,2]. The use of ACN-rich extracts as food colorants has increased in recent years due to potential behavioral concerns associated with the consumption of artificial dyes [3,4]. Also, interest in ACN consumption has grown due to their potential bioactive and health-promoting properties [5]. However, from an industrial point of view, the application of ACN-rich extracts as food colorants is restricted due to limited long-term stability and color expression [1]. Additionally, common components in the food matrix such as ascorbic acid can bleach ACNs, resulting in their degradation [6]. Several mechanisms have been proposed for the stabilization of ACNs in foods such as copigmentation with phenolic compounds [7], complexation with proteins [8], encapsulation within polysaccharides [9], and chelation with metals [10]. 

Studies have shown that prolonged interaction of ACNs with hydroxycinnamic acids [7], acetone [11], or pyruvic acid (PA) [12] can result in the formation of ACN-derived pigments called pyranoanthocyanins (PACNs). This process occurs during winemaking as a result of the interaction between ACNs and yeast metabolites [13,14]. These ACN-derived pigments can express color across all pH values [11] and have better long-term storage stability [6,11]. Moreover, PACNs showed enhanced resistance to bleaching by ascorbic acid [6] and sulfur dioxide [11,15]. These pigments are formed through the cycloaddition reaction of a reactive cofactor on position C4 and the 5-OH group of the ACN molecule, resulting in the formation of an additional pyran ring [16] and consequently, in the unavailability of C4 to participate in degradation reactions [17]. Studies on the synthesis pathways of different PACNs from ACN-rich extracts have shown that carboxy-PACNs can result from the reaction with PA, hydroxyphenyl-PACNs from the reaction with hydroxycinnamic acids, and methyl-PACNs from the reaction with acetone [11,18]. Due to this formation process, ACNs with substitutions on position C5 cannot form PACNs, thus excluding many pigment sources commonly used in the food industry from being PACN precursors. ACN sources commonly used in the food industry capable of forming PACNs include black carrot [7,19], elderberries [7], and some grape extracts [20]. 

Although more stable, PACNs are hard to find in nature with limited quantities reported in onions [21] and strawberries [22]. Additionally, PACNs can be found in a small number of foods such as wine [18], sumac [23], fruit wines [24], and fruit juices after extended periods of time [19]. Most studies have focused on the occurrence and identification of PACNs [18,19,20,25], their stability [6,11], and their unique color characteristics [26]. However, little has been reported on the optimization of their formation. Previous studies reported that PACN formation efficiency is increased with 1-6 di-glycosylated ACNs [12], the removal of ACN aromatic acylating groups [27], solution pH ~3.0 [28], as well as higher incubation temperatures [29] and molar cofactor ratios [28,29]. In addition, studies comparing different cofactors showed greater PACN yields with caffeic acid (CA) than with PA [27]. However, the impact on PACN formation efficiency of other cofactors, such as acetone, and modifications dependent on cofactor type and ACN structure are still underreported. This information is important if PACNs are to be used by the food industry as it would help in the development of more stable, naturally derived colorants. Therefore, the objective of this study was to determine the effect of the chemical structure of the cofactor and ACN on PACN formation efficiency under accelerated conditions (pH 3.1, ACN to cofactor molar ratio of 1:30, incubation at 45 °C).

## 2. Results

### 2.1. Evaluation of Pyranoanthocyanin Formation Efficiency Using Different Types of Cofactors

**Figure 1:**
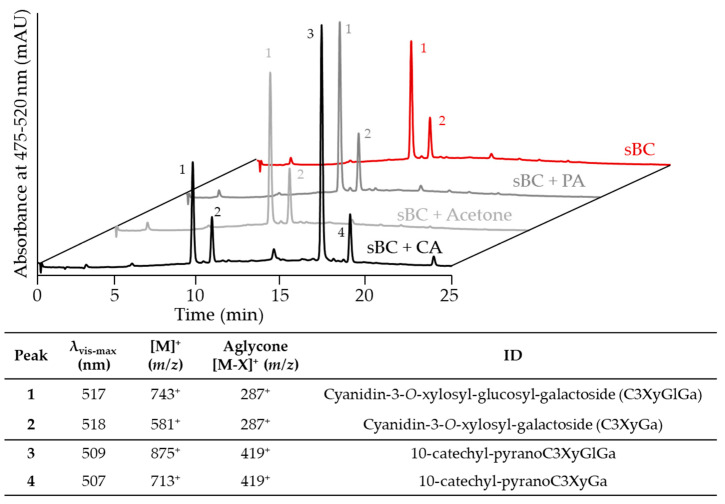
Comparing the PACN formation after 72 h of anthocyanin (saponified black carrot, sBC) incubation with caffeic acid (CA), acetone, or pyruvic acid (PA). Chromatograms show the max plot in the 475–520 nm range. Table shows the wavelength of maximum absorption in the visible range (λ_vis-max_), mass per charge ratio (*m*/*z*) of the main ion and its aglycone, and tentative identity (ID).

CA, acetone, and PA were tested as cofactors for the formation of PACNs from cyanidin-glycosides obtained from saponified black carrot ACN extracts (sBC) with results shown in Figure 1. The ACN extract was mainly composed of cyanidin-3-*O*-xylosyl-glucosyl-galactoside (peak 1, C3XyGlGa, ~64% of total area at 475–520 nm) and cyanidin-3-*O*-xylosyl-galactoside (peak 2, C3XyGa, ~34% of total area at 475–520 nm). The 475–520 nm max plot chromatogram was used because it accounted for the λ_vis-max_ of all pigments evaluated in this study. After incubation with CA, two new peaks were detected in the PDA chromatogram. Their hypsochromic shift of λ_vis-max_ compared to the precursor ACNs, later retention times, and MS spectra revealed that these new peaks corresponded to 10-catechyl-PACNs derived from the ACNs previously identified in sBC. Consequently, peak 3 was ascribed to 10-catechyl-pyranocyanidin-3-*O*-xylosyl-glucosyl-galactoside and peak 4 to 10-catechyl-pyranocyanidin-3-*O*-xylosyl-galactoside. No new peaks were detected in the cofactor-free control and samples with acetone and PA after 72 h of incubation. 

**Figure 2:**
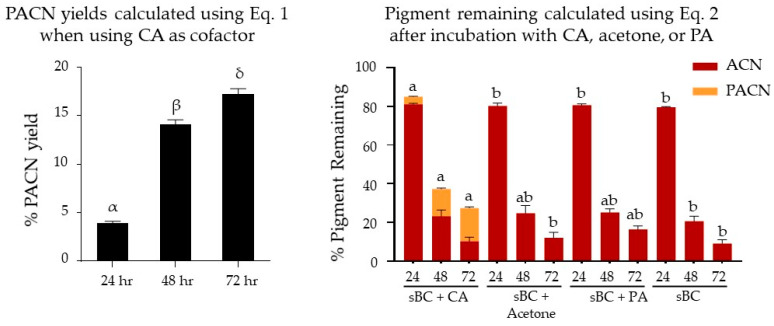
Pyranoanthocyanin yield (%PACN) with caffeic acid (CA) and pigment remaining (%) after incubation with different cofactors for 24, 48, and 72 h at 45 °C with cyanidin-glycosides (from saponified black carrot, sBC). Different Greek letters show significant differences among time points at a 0.05 level. Different letters show statistically significant differences among cofactors at the same time point at a 0.05 level. Results are expressed as means ± standard error (*n* = 3). ACN: anthocyanin, PA: pyruvic acid.

Yields of PACNs, calculated as percentages compared to the initial ACN content using Equation (1), increased from 3.9 ± 0.2% at 24 h to 14.1 ± 0.5% and 17.2 ± 0.6% after 48 and 72 h of incubation with CA at 45 °C, respectively (Figure 2). Based on the peak areas calculated at each time point using Equation (3), PACNs comprised 4.6 ± 0.2, 38.4 ± 3.5, and 63.5 ± 4.2% of the total pigment at 24, 48, and 72 h, respectively. No PACNs were detected in samples incubated with acetone or PA. The pigment remaining after 72 h of incubation was the highest in the samples with CA (27.4 ± 2.6%). This content was higher than the one observed in samples with PA (16.4 ± 1.8%), but not significantly so (*p* = 0.06). Additionally, the pigment remaining in samples with CA was significantly higher than the ones observed in cofactor-free control samples (9.1 ± 1.9%, *p* < 0.01) and samples with acetone (12.1 ± 2.8%, *p* < 0.01).

### 2.2. Evaluation of Pyranoanthocyanin Formation Efficiency Using Different Hydroxycinnamic Acids

**Figure 3:**
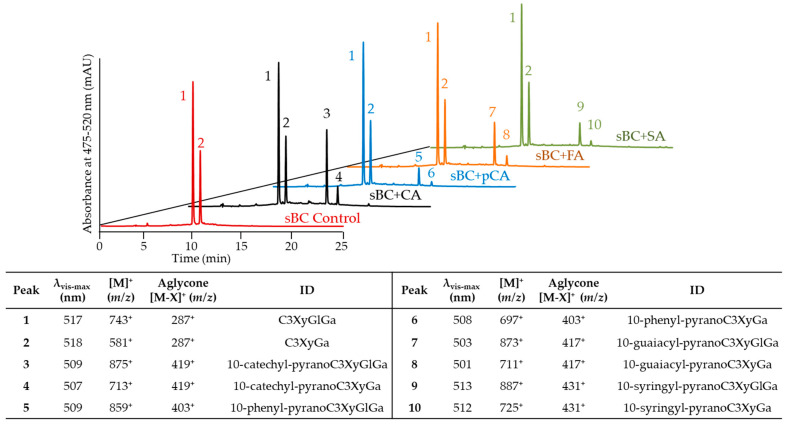
Comparing the pyranoanthocyanin formation after 72 h of anthocyanin (saponified black carrot, sBC) incubation with caffeic acid (CA), *p*-coumaric acid (pCA), ferulic acid (FA), and sinapic acid (SA). Chromatograms show the max plot in the 475–520 nm range. Table shows the wavelength of maximum absorption in the visible range (λ_vis-max_), mass-per-charge ratio (*m*/*z*) of the main ion and its aglycone, and tentative identity (ID). C3XyGlGa: cyanidin-3-*O*-xylosyl-glucosyl-galactoside, C3XyGa: cyanidin-3-*O*-xylosyl-galactoside.

After incubation for up to 72 h, CA was the only cofactor forming PACN under our experimental conditions, strongly suggesting that the chemical structure of the cofactor can impact PACN yields. Studies have shown different reactivity rates of hydroxycinnamic acids with ACNs or PACNs resulting in the formation of new pigments [30,31]. In these studies, it was hypothesized that the nucleophilic nature of the hydroxycinnamic acid impacted its reactivity [31], with di- and trisubstituted ones more reactive than monosubstituted ones [30]. Therefore, we hypothesized that minor structural differences among hydroxycinnamic acids (CA, *p*-coumaric acid (pCA), ferulic acid (FA), and sinapic acid (SA)) may also affect PACN yields under accelerated conditions. Analysis with UHPLC-PDA coupled to a tandem mass spectrometer with electrospray ionization (ESI-MS/MS) showed that the identities of peaks 1–4 (deriving from incubation with CA) in Figure 3 were consistent with the ones previously reported in Figure 1. The incubation with the other hydroxycinnamic acids resulted in the formation of new compounds denoted by peaks 5–10 with later retention times and hypsochromic effects on λ_vis-max_ (Figure 3). These characteristics along with the [M]^+^ and [M–X]^+^ values were consistent with hydroxyphenyl-PACNs derived from non-acylated ACNs present in sBC. Peaks 5 and 6 were attributed to 10-phenyl-pyranocyanidin-3-*O*-xylosyl-glucosyl-galactoside and 10-phenyl-pyranocyanidin-3-*O*-xylosyl-galactoside, respectively. Similarly, peaks 7 and 8 were recognized as 10-guaiacyl derivatives and peaks 9 and 10 as 10-syringyl derivatives (Figure 3).

**Figure 4:**
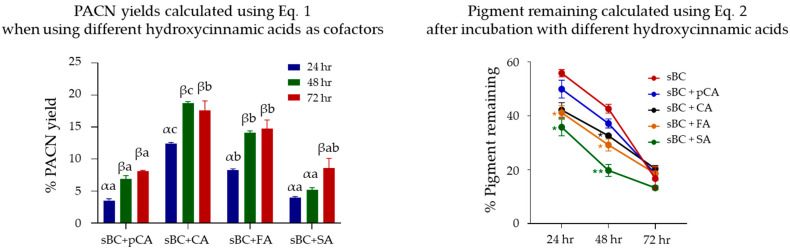
Pyranoanthocyanin yield (%PACN) with different hydroxycinnamic acids and pigment remaining (%) after incubation for 24, 48, and 72 h at 45 °C with cyanidin-glycosides (from saponified black carrot, sBC). Different Greek letters show significant differences among time points at a 0.05 level. Different letters show significant differences among cofactors at the same time point at a 0.05 level. Asterisks (*) and (**) indicate significant differences against the cofactor-free control (sBC) at a 0.05 and 0.01 level, respectively. Results are expressed as means ± standard error (*n* = 3). pCA: *p*-coumaric acid. CA: caffeic acid, FA: ferulic acid, SA: sinapic acid, Eq: equation.

Yields calculated using Equation (1) and displayed in Figure 4 showed that after 24 h of incubation, PACN yield with CA (12.5 ± 0.2%) was significantly greater than with FA (8.3 ± 0.2%, *p* < 0.01). Moreover, FA had significantly higher yields than pCA (3.5 ± 0.3%, *p* < 0.01) and SA (4.0 ± 0.2%, *p* < 0.01), with no significant differences among these last two cofactors (*p* > 0.05). After 48 h of incubation, the same efficiency pattern was observed. After 72 h of incubation, CA and FA were significantly more efficient than pCA (*p* = 0.0476 and *p* = 0.0246, respectively). Yields with SA were lower than with CA and FA, but not significantly so (*p* > 0.05 for both CA and FA). When analyzing the percent of pigment remaining, results in Figure 4 showed that after 24 h, incubation with FA (41.2 ± 2.6%) or SA (35.8 ± 3.2%) resulted in a significantly lower pigment remaining (*p* = 0.0367 and *p* = 0.3350, respectively) compared to the cofactor-free control (55.8 ± 1.4%). However, these differences were not significant after 72 h of incubation (*p* > 0.05).

### 2.3. Evaluation of Pyranoanthocyanin Formation Efficiency Using Different Anthocyanins

**Figure 5:**
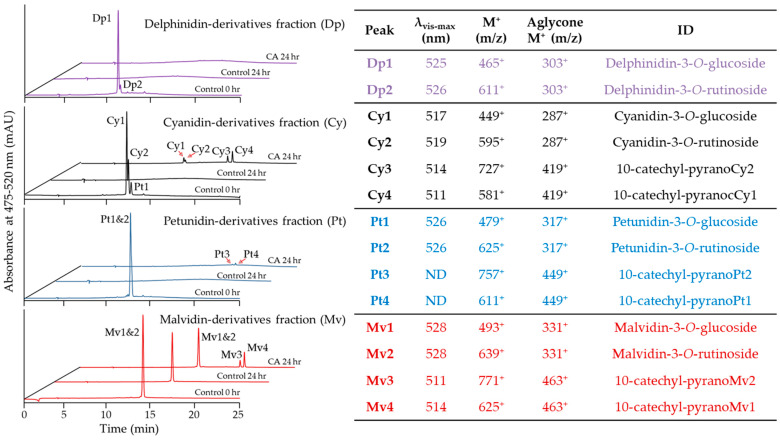
Anthocyanins from fractions with different aglycones and PACN formation after 24 h of 45 °C incubation with caffeic acid (CA). Chromatograms show the max plot in the 475–520 nm range. Table shows the wavelength of maximum absorption in the visible range (λ_vis-max_), mass-per-charge ratio (*m*/*z*) of the main ion and its aglycone, and tentative identity (ID).

Minor structural differences among hydroxycinnamic acids impacted PACN yields. Therefore, we hypothesized that minor structural differences among ACNs could also impact PACN yields. *Berberis boliviana* was chosen as the pigment source for this comparison because it contains simple ACNs deriving from four different aglycones in similar proportions [32]. Results confirmed the presence of delphinidin, cyanidin, petunidin, and malvidin derivatives in the *Berberis boliviana* ACN extract used in this study. In addition, results showed the presence of two glycosylation patterns consistent with glucoside, denoted by a neutral loss of 162 *m*/*z*, and rutinoside, denoted by a neutral loss of 308 *m/z* (Figure 5). After fractionation using semi-preparative HPLC-PDA, four fractions were obtained each with glucoside and rutinoside derivatives of different ACN aglycones. Further UHPLC-PDA-ESI-MS/MS analyses were carried out to identify the individual ACNs in each fraction, and their identities were compared against previous literature. The pigments in the delphinidin-derivatives fraction (Dp) were delphinidin-3-*O*-glucoside (Dp1, ~91%) and delphinidin-3-*O*-rutinoside (Dp2, ~7%), pigments in the cyanidin-derivatives fraction (Cy) were cyanidin-3-*O*-glucoside (Cy1, ~65%) and cyanidin-3-*O*-rutinoside (Cy2, ~27%) with a minor content of petunidin-3-*O*-glucoside (Pt1, ~8%), pigments in the petunidin-derivatives fraction (Pt) were petunidin-3-*O*-glucoside (Pt1, ~73%) and petunidin-3-*O*-rutinoside (Pt2, ~27%), and pigments in the malvidin-derivatives fraction (Mv) were malvidin-3-*O*-glucoside (Mv1, ~77%) and malvidin-3-*O*-rutinoside (Mv2, ~22%). 

After 24 h incubation with CA, fractions with cyanidin-, petunidin-, or malvidin-derived ACNs developed peaks with later retention times and hypsochromic λ_vis-max_. UHPLC-PDA-ESI-MS/MS analyses showed that these new peaks were consistent with 10-catechyl derivatives of the ACNs present in each fraction. Indeed, peaks Cy3 and Cy4 were named 10-catechyl-pyranocyanidin derivatives, peaks Pt3 and Pt4 10-catechyl-pyranopetunidin derivatives, and peaks Mv3 and Mv4 10-catechyl-pyranomalvidin derivatives (Figure 5). It is worth noting that incubation of delphinidin-derivatives with CA yielded no PACNs under our experimental conditions. 

**Figure 6:**
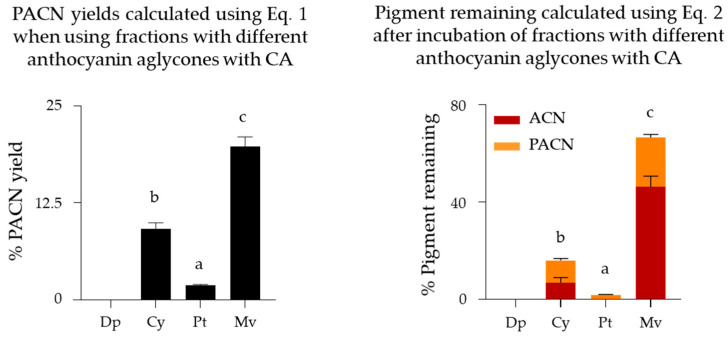
Pyranoanthocyanin yield (%PACN) and pigment remaining (%) after incubation of anthocyanin fractions with different aglycones with caffeic acid (CA) for 24 h at 45 °C. Different letters show significant differences among different fractions at a 0.05 level. Results are expressed as mean ± standard error (*n* = 3). ACN: anthocyanin, Dp: delphinidin-derivatives fraction, Cy: cyanidin-derivatives fraction, Pt: petunidin-derivatives fractions, Mv: malvidin-derivatives fraction, Eq: equation.

The highest PACN yield (Figure 6) was observed for malvidin-derivatives followed by cyanidin-derivatives (19.8 ± 1.2% and 9.2 ± 0.8%, respectively, after 24 h of incubation, *p* < 0.01). Furthermore, using malvidin-derivatives not only yielded more PACNs but also resulted in a significantly higher percent of pigment remaining after incubation (64.1 ± 5.2%, *p* < 0.01), followed by cyanidin-derivatives (16.0 ± 2.2%) and then petunidin-derivatives (1.9 ± 0.1%). 

When analyzing the relative content of PACNs with a rutinoside substitution as a percentage of total PACNs (Figure 5), this content was 36.1 ± 0.2% in the cyanidin-derivatives fraction, 32.4 ± 1.1% in the petunidin-derivatives fraction, and 31.4 ± 0.4% in the malvidin-derivatives fraction. These values were higher than the ACN-3-*O*-rutinoside proportion in the original ACN extract analyzed in our preliminary analysis (data not shown).

## 3. Discussion

Under accelerated formation conditions (pH 3.1, 1:30 ACN to cofactor molar ratio, and 45 °C incubation temperature), PACNs were detected in as little as 24 h with yields up to 19.8% using malvidin-derived ACNs and CA as the cofactor. These yields were at least six times higher than those reported for malvidin-3-*O*-glucoside with CA after four months using wine-like systems (pH 3.2, ~1:2 ACN to cofactor molar ratio, and incubation at 15 °C) [31]. Additionally, the PACN yields obtained under our experimental conditions were at least five times higher than those reported for malvidin-3-*O*-glucoside with CA or FA and 20 times higher than those reported with pCA in synthetic grape mediums after 14 days (pH 3.5, ~1:0.8 ACN-to-cofactor molar ratio, and incubation at 30 °C) [33]. The higher yields observed in our study could be attributed to the use of conditions previously reported to accelerate PACN formation [27,28,29]. However, our results demonstrated that other factors such as the cofactor chemical structure and the ACN type also played a key role in PACN formation efficiency under accelerated formation conditions.

The PACN yields observed in this study using cyanidin-derived ACNs and CA were comparable to previous reports evaluating yields under similar conditions. Straathof and Giusti reported PACN yields of ~20% after 24 h of incubation at 65 °C using elderberry ACNs with CA [29], which was higher than those obtained under our experimental conditions when comparing different hydroxycinnamic acids as cofactors. This higher yield could be attributed to higher incubation temperatures and molar cofactor ratio used (1:50) as well as the different glycosylation patterns of elderberry ACNs. To evaluate potential differences in PACN yields as a result of the use of different cofactors and ACN types, 45 °C instead of 65 °C was selected to reduce pigment degradation as reported by Straathof and Giusti [29] at temperatures of 65 °C or above. In addition, our PACN yields after 72 h of incubation were similar to the ones reported by Zhu and Giusti after seven days of incubation at 25 °C [27]. In that study, incubation for seven additional weeks led to higher PACN yields (~71–85%) that were not obtained under the accelerated conditions used in our experiment. 

To compare different cofactors, black carrot was selected as a pigment source because its ACNs do not have glycosylations on position C5 [7], and this allowed for the cycloaddition reaction with a reactive adduct resulting in the formation of a pyran ring between the C4 and 5-OH groups of the ACN molecule [16]. Moreover, the tri- and di-glycosylation patterns at C3 of these ACNs allowed for better solubility of their derived PACNs, preventing their precipitation as observed in our preliminary studies with ACNs from other sources. As sinapoyl acylating groups have been shown to decrease PACN formation efficiency [27], alkaline hydrolysis was carried out to remove the aromatic acylating moiety.

Among the cofactors tested (CA, acetone, and PA), CA was the only one that produced PACNs under our experimental conditions. Cofactors with an aromatic ring, such as hydroxycinnamic acids, can copigment with ACNs [7,34]. Through intermolecular interactions, the aromatic cofactor is brought in closer proximity to the ACN, which can stabilize the chromophore during the incubation period and increase the likelihood of a reaction [27,28,35]. Copigments must contain a benzene ring for π-π interactions with ACNs to take place [36]. Aliphatic cofactors such as PA and acetone are not expected to copigment with ACNs, which could reduce their interaction with ACNs thus resulting in a lower PACN yield compared to CA [27]. Previous studies have found PACN formation yields 3.5 to 4 times greater with CA as a cofactor than with PA [27,28]. While we did not detect PACNs forming from PA even after 72 h, the longer incubation periods of 42 and 56 days and greater molar ratios of PA (1:100 and 1:200) used in those studies may have compensated for the less efficient cofactor–ACN interaction to facilitate PACN formation [27,28]. 

CA was the most efficient cofactor even when compared to other hydroxycinnamic acids, yielding the highest amount of PACNs after only 24 h of incubation (CA > FA > SA = pCA). However, after 72 h, no statistical differences were observed in the PACN yield between samples incubated with CA, FA, or SA, although yields with SA were lower and not statistically different than yields with pCA. CA and FA are widely distributed in natural sources. CA is abundant in plants such as aronia berries [37] and black carrots [38], and FA is found in plant cell walls and can be released into a solution by alkaline hydrolysis [39]. Therefore, the saponification process we used to remove the acylating group in the ACN molecule making it more predisposed for PACN formation [27] can, at the same time, release one of the most efficient cofactors into a solution. 

The efficiency pattern of hydroxycinnamic acids observed in our results is similar to the one reported in wine-like model solutions [31,40], despite differences in cofactor molar ratios, incubation temperatures, and PACN yields. Additionally, similar reactivity patterns were observed for hydroxycinnamic acids during the formation of portisins [30]. It has been hypothesized that bond formation between C4 of the ACN and the alpha carbon of the hydroxycinnamic acid is affected by the nucleophilic nature of the cofactor [40]. The stronger nucleophilic nature of CA as a result of the two hydroxyl substitutions in the phenolic ring [41] could explain its higher PACN formation efficiency. Additionally, steric hindrance impacts PACN formation efficiency [12,26]; this hindrance may explain the higher PACN yields with FA over SA as cofactors. 

When evaluating the impact of the ACN aglycone on PACN formation using CA as a cofactor, malvidin was the most efficient followed by cyanidin (malvidin > cyanidin > petunidin). Malvidin-3-*O*-glucoside has been shown to have stronger copigmentation interactions with CA than other ACN-3-*O*-glucosides [34]. As copigmentation with the cofactor may facilitate PACN formation [27], the increased interactions may contribute to greater PACN formation rates with malvidin-derived ACNs. This stronger copigmentation with CA may also help to explain the significantly higher pigment remaining after incubation for 24 h. Additionally, the higher content of ACNs remaining in the malvidin fraction after 24 h incubation (~45% of initial pigment) may indicate that greater PACN yields may be achieved with longer incubation times. Results also showed very small amounts of PACNs in samples with petunidin-derived pigments (~1.9%) and no PACNs detected in samples with delphinidin-derivatives. Furthermore, after incubation at 45 °C for 24 h, there was little to no pigment remaining in these samples; this negates the possibility of PACN formation with increased incubation times. Delphinidin’s and petunidin’s propensity to degrade during prolonged heating [42,43] may explain the absence or reduced formation of PACNs in these samples. The significantly higher yields of malvidin- and cyanidin-derived PACNs may explain why most reports have especially identified these derivatives [11,14,16,19,25,44,45].

The ACN fractions used to compare the impact of the aglycone type contained two glycosylation patterns (glucoside and rutinoside). Glycosylation patterns have been reported to impact PACN yields with cyanidin-3-*O*-rutinoside yielding more PACN than cyanidin-3-*O*-glucoside when incubated with PA at 25 °C [12]. This observation was consistent with our results; indeed ACN-3-*O*-rutinosides seemed to form PACNs more efficiently than their glucoside counterparts denoted by an increased content of rutinoside-derivatives in the PACN fraction after incubation with CA (from ~27% to ~37% in the Cy fraction, from ~27% to ~33% in the Pt fraction, and from ~22% to ~32% in the Mv fraction). Despite having similar glucoside to rutinoside proportions, PACN yields with CA were significantly different between the Cy, Pt, and Mv fractions. This difference suggests that the impact of the glycosylation may be secondary to the impact of the aglycone type on PACN yield, possibly due to the aglycone influence on stability and copigmentation ability. 

## 4. Materials and Methods

### 4.1. Plant Material, Chemical, and Reagents

*Berberis boliviana* freeze-dried berries were generously donated by Carla del Carpio from Universidad Nacional de San Antonio Abad del Cusco (UNSAAC, Cusco, Peru). A commercial black carrot color powder (*Daucus carota*) was provided by D.D. Williamson (Louisville, KY, USA). CA, FA, SA, and PA were purchased from Sigma-Aldrich (St. Louis, MO, USA). Acetone, chloroform, acetonitrile, UHPLC-grade water, pCA, sodium benzoate, and sodium hydroxide were obtained from Fisher Scientific (Pittsburgh, PA, USA). Potassium sorbate was obtained from Spectrum (New Brunswick, NJ, USA). All other reagents and solvents were of at least analytical or HPLC grade unless otherwise indicated. 

### 4.2. Anthocyanin Preparation 

ACN extraction was carried out following the methodology described by Rodriguez-Saona and Wrolstad [46] with minor modifications. Black carrot color powders were prepared for extraction by reconstituting them in water. *Berberis boliviana* berries were deseeded manually, soaked in water, and frozen with liquid nitrogen before extraction. Then, the frozen berries and reconstituted black carrot color powder were each blended with 0.01% HCl acetone (*v*/*v*) using a Waring laboratory blender. The slurry was filtered through an N° 4 Whatman filter paper, and the cake was re-extracted using 0.01% HCl aqueous acetone (70% *v*/*v*) until a faint pink solution was obtained. The filtrate was then partitioned using chloroform for a final acetone:chloroform proportion of 1:2 (*v*/*v*) in a separatory funnel and stored overnight at 4 °C. The upper aqueous layer containing ACNs and other phenolic compounds was collected, and residual solvents were evaporated using a Büchi rotavapor at 45 °C (Büchi, Flawil, Switzerland). 

### 4.3. Saponification of Black Carrot Anthocyanins 

Alkaline hydrolysis of acylated ACNs from black carrot was carried out to remove the acylation moiety in their structure following the methodology described by Giusti and Wrolstad [47]. Briefly, solutions rich in black carrot ACNs were mixed with 10% KOH (*w*/*v*) in a proportion of 1:10 and left to stand for 8 min in the dark at room temperature, after which the pH was neutralized using 2N HCl. Neutralized, saponified extracts were subjected to solid phase extraction (SPE) following the methodology described in Section 4.4. The obtained semi-purified, sBC was later used for comparison of cofactor efficiency. 

### 4.4. Pigment Semi-Purification

ACN semi-purification was performed using SPE following the methodology described by Rodriguez-Saona and Wrolstad [46] with minor modifications. Briefly, crude ACN extracts were diluted in acidified water prior to SPE using Waters Sep-pak C18 cartridges (Waters, Milford, MA, USA). Cartridges were activated with methanol and then washed with 0.01% HCl (*v*/*v*) acidified water, after which the crude extract was applied to the cartridge. Salts, sugars, and organic acids were removed using two volumes of acidified water followed by washing with three to four volumes of ethyl acetate to remove less polar phenolics. Semi-purified pigments were eluted from the column using 0.01% HCl (*v/v*) acidified methanol. The solvent was then evaporated using a rotavapor at 45 °C, and pigments were resolubilized in acidified water and stored under refrigeration until further use. 

### 4.5. Fractionation of Different Anthocyanins 

Fractions of different ACNs from semi-purified *Berberis boliviana* extract prepared in Section 4.4 were obtained using semi-preparative HPLC-PDA (Shimadzu, Columbia, MD, USA). Reverse phase chromatographic separation was achieved using a Synergi 4 µm Max-RP 80 Å column of 250 × 21.2 mm dimensions (Phenomenex, Torrance, CA, USA) and a binary solvent system composed of A: 4.5% formic acid in water (*v/v*) and B: acetonitrile at a flow rate of 10 mL/min. Elution gradient started at 10–20% B from 0 to 20 min, 20–40% B from 20 to 21 min, and 40% B from 21 to 24 min. Collected fractions were concentrated using SPE to remove formic acid and acetonitrile, and residual methanol was evaporated using a rotavapor at 45 °C. Isolated fractions were resolubilized with acidified water and stored under refrigeration until further use. 

### 4.6. Anthocyanin and Pyranoanthoycanin Identification 

ACN and PACN tentative identification were carried out using a Nexera-i-LC-2040 3D ultra-high performance liquid chromatograph (Shimadzu, Columbia, MD, USA) coupled with an LCMS-8040 triple quadrupole mass spectrometer with an ESI interface (Shimadzu, Columbia, MD, USA). Chromatographic separation was achieved using a Synergi 4 µm Max RP-80 Å 250 × 4.6 mm column (Phenomenex, Torrance, CA, USA) and a binary solvent system consisting of A: 4.5% formic acid and B: acetonitrile with a gradient of 5–45% B from 0 to 20 min and 45% B from 20 to 25 min. After chromatographic separation, a volume of approximately 0.2 mL/min was diverted into the MS/MS for analyses. Tentative identification of pigments was carried out based on their elution time, UV-Vis spectral characteristics, and corresponding *m*/*z* using ESI-MS/MS. Mass spectrometry analyses were conducted under positive ion mode with 1.5 L/min nebulizing gas flow, 15 L/min drying gas, a desolvation gas temperature of 230 °C, and collision energy of -35 eV. Spectral data were acquired using total ion scan mode from *m*/*z* 100 to 1000 and precursor ion scan mode for the most common six anthocyanidins in nature (271, 287, 301, 303, 317, and 331 *m*/*z*). Identification of new colored compounds was carried out using precursor ion scans with the expected PACN aglycone *m*/*z* listed in the tables in Figure 1, Figure 3 and Figure 5 for each experimental section. Data analysis and interpretation were performed using Lab Solutions Software Ver. 1 (Shimadzu, Columbia, MD, USA).

### 4.7. Monomeric Anthocyanin Quantitation

Monomeric ACNs were quantified using the pH differential method [48]. Briefly, semi-purified ACN extracts or isolated ACN fractions were diluted in 0.025 M potassium chloride buffer at pH 1 or 0.4 M sodium acetate buffer at pH 4.5. The difference of absorbance at their respective λ_vis-max_ and 700 nm was measured using a SpectraMax M2 plate reader (Molecular Devices, Sunnyvale, CA, USA). The monomeric ACN content in sBC was expressed as cyanidin-3-*O*-glucoside equivalents while the content in each ACN fraction from *Berberis boliviana* was expressed in equivalents of its most abundant ACN (delphinidin-3-*O*-glucoside, cyanidin-3-*O*-glucoside, petunidin-3-*O*-glucoside, or malvidin-3-*O*-glucoside) using the corresponding molecular weight and molar absorptivities reported in the literature [48,49]. 

### 4.8. Pyranoanthocyanin Formation—Comparing Different Types of Cofactors

PACN formation was carried out following the methodology described by Straathof and Giusti [29] with minor modifications. Briefly, stock solutions of sBC and three different types of cofactors (PA, acetone, and CA) were prepared using pH 3.1 acidified water containing 0.1% potassium sorbate (*w*/*v*) and 0.1% sodium benzoate (*w*/*v*). Stock solutions of pigment and cofactor were mixed and diluted to a pigment concentration of 80 µM and a cofactor concentration of 2.4 mM (1:30 pigment to cofactor molar ratio). The final solution was adjusted to pH 3.1 ± 0.05 with 1 M NaOH and 2N HCl when needed. Samples were placed into HPLC vials and stored in the dark in a Roto-Therm™ H2020 benchtop incubator (Benchmark Scientific, Edison, NJ, USA) set at 45 °C. Every 24 h, the vial was removed, run on the HPLC, and returned to the incubator for additional incubation, up to 72 h total. 

### 4.9. Pyranoanthocyanin Formation—Comparing Different Hydroxycinnamic Acids

The effects of minor structural differences among different hydroxycinnamic acids on PACN formation were evaluated following the methodology described in Section 4.8. The sBC extract was used as the pigment source, and four different hydroxycinnamic acids (CA, pCA, FA, and SA) were tested as cofactors at equivalent 1:30 ACN to cofactor molar ratios and incubated at 45 °C in pH 3.1 solution in the dark.

### 4.10. Pyranoanthocyanin Formation—Comparing Different Aglycones

The effect of the aglycone structure on the formation efficiency of PACNs was evaluated using ACN-rich fractions containing a different aglycone and the cofactor identified as producing the highest PACN yield (in this case, CA) following the methodology described in Section 4.8. *Berberis boliviana* was selected as the pigment source because it contains four of the six most common ACNs found in nature in relatively similar proportions with glycosylations (either glucose or rutinose) only on position C3 [32]. CA was used as a cofactor at a 1:30 ACN to cofactor molar ratio, and samples were incubated at 45 °C in pH 3.1 solution in the dark.

### 4.11. Monitoring Pyranoanthocyanin Formation and Anthocyanin Changes Over Time 

Total pigment content and PACN formation were monitored during incubation using HPLC-PDA (Shimadzu, Columbia, MD, USA) and calculated using the areas under the curve (AUC) of the compounds of interest. The system consisted of two LC-20AD pumps, a CBM-20A controller, a SIL-20AC refrigerated autosampler, an SPD-M20A PDA detector, and a CTO-20A column oven. Chromatographic separation was achieved using the same column, solvents, and gradient parameters as in Section 4.6. 

PACN formation yield was calculated using Equation (1):

(1) $$PACN\;formation\;yield\;\left(\%\right)=\frac{\left(AUC_{475-520\;nm}\;PACNs\;at\;t_{n}\right)}{\left(AUC_{475-520\;nm}\;ACNs\;at\;t_{0}\right)}*100$$

The percent of pigment remaining was calculated using Equation (2):

(2) $$Percent\;of\;Pigment\;Remaining=\frac{\left(AUC_{475-520\;nm}\;ACNs+PACNs\;at\;t_{n}\right)}{\left(AUC_{475-520\;nm\;}ACNs\;at\;t_{0}\right)}*100$$

The content of PACNs, as the percentage of total pigment at a given time point, was calculated using Equation (3):

(3) $$Percent\;of\;PACN\;to\;total\;pigment\;\left(\%\right)=\frac{\left(AUC_{475-520\;\mathrm{nm}}\;PACNs\;at\;t_{n}\right)}{\left(AUC_{475-520\;\mathrm{nm}}\;ACNs+PACNs\;at\;t_{n}\right)}*100$$

### 4.12. Statistical Evaluation of Data

Data from each replication were collected from duplicate samples and experiments were conducted in triplicate. Data were expressed as mean ± standard error of means. Differences among treatments were analyzed using a one-way analysis of variance (ANOVA) with Bonferroni post-hoc tests. Analyses were conducted using GraphPad Prism (GraphPad Software Inc., La Jolla, CA, USA). A *p*-value lower than 0.05 was considered significant. 

## 5. Conclusions

The chemical structure of the cofactor and ACN type impacted PACN yields with significant differences detected after only 24 h of incubation. CA and FA were the most efficient cofactors with similar PACN yields when using cyanidin-glycosides from sBC as the ACN source (~19% and ~14%, respectively). Among the different types of aglycones tested, malvidin-glycosides were the most efficient for PACN formation with CA (~20% after 24 h). Overall, this research demonstrated that cyanidin- or malvidin-derived ACNs in combination with CA or FA can produce high amounts of PACNs under accelerated formation conditions. These results highlight the importance of the ACN source and cofactor selection for the efficient production of PACNs. Given their scarcity in nature, this efficient production could facilitate their use by the industry as naturally derived colorants with increased stability. 

## Data Availability

The data that supports the findings of this study are available from the corresponding author upon reasonable request.
